# Physician Perspectives on Factors That Influence Patients’ Choice Between the NHS and Private Healthcare: A Qualitative Study

**DOI:** 10.7759/cureus.78331

**Published:** 2025-02-01

**Authors:** Vigneshwar Veerappan, Shraddha Burway, Andrea Saji, Pranit Sukumar, William Laughey

**Affiliations:** 1 Psychiatry and Behavioural Sciences, Bradford Teaching Hospitals NHS Foundation Trust, Bradford, GBR; 2 Medicine, Hull York Medical School, York, GBR; 3 Medicine, Yeovil District Hospital NHS Foundation Trust, Yeovil, GBR; 4 Medicine, Hull University Teaching Hospital NHS Trust, Hull, GBR; 5 Medical Education, Hull York Medical School, York, GBR

**Keywords:** healthcare, healthcare governance, health policy, health service, patient-centred care

## Abstract

Introduction

The National Health Service (NHS) is the primary provider of healthcare in the UK. Due to several reasons, further exacerbated by the COVID-19 pandemic, the NHS has struggled to meet the healthcare needs of the public. In this healthcare climate, the private sector holds unique opportunities and hurdles. While the relationship between the NHS and the private healthcare sector has been studied, there is a dearth of literature on how patients perceive this relationship and what factors influence them to choose between the two. The aim of this study was to qualitatively identify factors that influence a patient’s choice between the NHS and private healthcare.

Methodology

Ten physicians (six general practitioners (GPs) and four secondary care consultants) were virtually interviewed. Interviews were semi-structured with a question stem, but the interview was open to interviewee-led digression. An iterative approach was taken, and Braun and Clarke’s six steps of thematic analysis were undertaken by researchers to analyse the data.

Results

Three global themes were identified: patient factors, physician factors, and service factors. Factors that influence patients’ choice between private healthcare or the NHS were largely the waiting time in the NHS and the control patients had in private healthcare. Other factors include the greater comfort, time and attention provided in private healthcare.

Conclusion

This study identified three global themes (patient, physician and service factors) from a physician’s perspective. However, these themes had significant overlap and their nuanced interactions warrant more in-depth study. While the study had several limitations, it provides a foundation for more studies that can examine the relationship between the NHS and the private sector from a patient’s perspective.

## Introduction

The National Health Service (NHS) serves as the primary healthcare provider in the United Kingdom (UK), delivering free services at the point of care [1]. Research has demonstrated that the NHS faces significant challenges in domains ranging from funding to workforce that prevent it from meeting the expectations of its patients [2,3]. Challenges such as workforce shortages have been acutely exacerbated by the COVID-19 pandemic [4]. Other contributing factors range from organisational issues such as underfunding to patient-specific concerns like discontinuity of care and inflexibility within the service structure [5,6].

The challenges faced by the NHS have opened up a larger avenue for the private healthcare sector to address the shortfalls and unmet healthcare needs of the population [7,8,9]. By shifting the burden of elective procedures away from the NHS, the private sector could enable the NHS to reallocate resources toward acute care, thereby reducing financial strain and increasing service capacity. This could benefit the overall healthcare system by reducing patient waiting lists [7,8]. However, an expanding private sector workforce also has the potential to divert resources away from the NHS [10].

Given the growth of the private sector, it is important to identify factors that influence the decision between opting for private or public healthcare. Studies from countries such as Italy and Israel have characterised determinants such as socioeconomic status, timeliness of care and hospitality as important determinants in choosing between private and public healthcare [11,12,13,14]. In the UK, approximately 10-12% of the population has access to private medical insurance and seeks private healthcare for various reasons [7]. Despite its importance, this complex relationship remains poorly understood, creating a notable gap in the literature within the UK. 

The aim of this study was to qualitatively identify the factors that influence a patient’s choice between the NHS and private healthcare. To achieve this, we conducted semi-structured interviews with clinicians working in both the NHS and private sectors, enabling us to gather insights from a larger patient population.

## Materials and methods

Research approach

We adopted a constructivist paradigm that highlights the subjective nature of reality and knowledge. We anticipated a relativist, qualitative approach that suits the how and why questions that this research poses. We selected Braun and Clarke’s approach to reflexive thematic analysis [15], particularly because it acknowledges the influence of the research team in data interpretation, which is in keeping with our chosen paradigm, and also because of its recognised utility for identifying latent themes with the data [16].

Setting and participants

Data were collected at Hull York Medical School (HYMS), during the time period November 2019 to October 2020, following institutional ethics board approval from HYMS (1937/2019). Two centres were selected to improve the transferability of results. Most researchers were senior medical students (VV, SB, AS and PS) and one was a GP and clinical tutor at HYMS (WL).

Recruitment was purposive, consisting of participants who could offer first-hand accounts of the NHS and private health sector in the UK from a physician's perspective. Participants were either consultants who work in the NHS and private sectors or GPs who refer to the NHS and private sectors. Participation was voluntary. Recruitment occurred via email and word of mouth.

Data collection and analysis

One researcher (VV) conducted all the one-to-one, semi-structured interviews. There was a question stem (see Appendix), but VV was open to digression made by the participants and to explore new lines of inquiry. The approach was iterative: questions were refined and added as data were analysed. Interviews were face-to-face or via video or audio call depending on the convenience of interviewees. Interviews were audio recorded and transcribed verbatim (by VV, SB, AS and PS). Braun and Clarke’s six steps of thematic analysis followed 1) familiarisation, 2) generating initial codes, 3) searching for themes, 4) reviewing themes, 5) defining and naming themes and 6) producing a report [15].

Within step 1, to enhance data familiarity, all researchers read and re-read at least one transcript, making notes in the margins of possible codes. Within step 2, VV and SB independently coded all transcripts, and all transcripts were double-coded by at least one other researcher who was not involved in the initial coding, aided by the sharing of coding documents via Google Drive. Within step 3, analysis of the pooled, coded data was conducted by two researchers (VV and SB) independently. Similar codes were collated into early sub-themes, and sub-themes were reviewed alongside one another to discern connections within the data. Any differences in generated sub-themes and themes were discussed and finalised between the two researchers. Within step 4, team discussions facilitated a review of early proposed themes and themes were defined and named as a group (step 5). VV produced a narrative report of results, which was discussed by all authors synchronously and asynchronously until a final report was agreed upon (step 6). While theoretical saturation as a concept was not formally applied to the dataset, it was noted that the last two interviews did not yield any unique codes or themes.

Reflexive considerations and setting

All researchers had some experience interacting with patients who had used private healthcare and the NHS. Through reflexive conversations, researchers discussed views and preconceptions about the role of private healthcare and how it can complement or conflict with the role of the NHS. Whilst a variety of views were held, no researchers were philosophically opposed to a private health option. All researchers work at HYMS a medium-sized medical school in the UK. All UK residents generally have access to NHS services. Some elect to use private healthcare services either through self-funding or through being a member of a private health insurance scheme, generally either self-funded or provided by their employer.

## Results

Demographics

In total, 10 participants were interviewed. Out of this, six participants were GPs, and four were consultants who work in the NHS and the private sector. Of the four consultants, there was one spinal neurosurgeon, one general surgeon, one ophthalmologist and one vascular surgeon. There were four females and six males overall.

Themes

**Figure 1:**
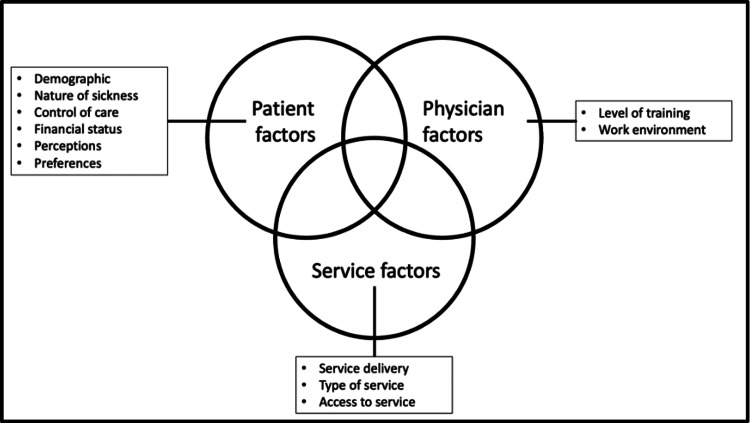
Pictorial representation of the global themes and sub-themes identified in this study Image credits: Vigneshwar Raj Veerappan

A total of three global themes were identified, as shown in Figure 1, namely, patient factors, physician factors and service factors. We have identified notable overlap between the three overall themes that together influence patients’ choice between the NHS and private healthcare. 

Theme 1: Patient Factors

GPs and surgeons highlighted patient demographics as an aspect that influenced their choice of healthcare service. Interviewees highlighted that patients of different ages may expect different things from their healthcare providers, ranging from the timeliness of the service to the actual physician involved.

"The older generation find it comforting to know that they will see a specialist, get some advice and they can go back for follow-up. The younger generation is happy to see a specialist promptly, it may not need a long-term follow-up."

Besides the patient’s inherent characteristics, the geographical location where they lived or sought healthcare was an equally influential sub-theme.

"Somewhere in London, for example, there’s probably a larger amount of private work than there is somewhere like Hull and more deprived areas."

The nature of the patient’s sickness was another sub-theme highlighted by the interviewees. For example, surgeons unanimously emphasised that the complexity of the patient’s case played a large role in what advice doctors would give regarding the management plan and choice of the healthcare sector. All of them agreed that more complex cases, where the surgery is high risk or the patient has multiple comorbidities, should be seen under NHS care as it tends to be more well-equipped than private healthcare. The multidisciplinary process in the NHS, in particular for complex patients, also offers a level of support for physicians, which may not be seen in private healthcare.

"I wouldn't perform any complex vascular surgical cases in the private sector…. So anything that needs arterial surgery, I wouldn't perform in the private sector these days. Post-op care, and management of complications, are all safer in the NHS."

Similarly, GPs re-iterated the importance of the nature of a patient’s sickness but took on a more holistic and longitudinal perspective, citing that the duration of a patient’s sickness (chronic versus acute) and the impact on their daily life are equally important. For example, GPs described the impact of a condition on a patient's career and activities of daily living instead of the complexity of a pathology itself.

Furthermore, interviewees stated that private healthcare provides patients with a level of control over their care that the NHS is unable to provide. This means patients are able to choose who they see and schedule convenient appointments with reduced risk of cancellations or delays.

"They feel a sense of being in control when it’s a private side because they are not being fed into a system where there are potentially waiting times, and they are waiting for a letter back as opposed to a private hospital where you know when the next appointment is, and how long the wait time is, and how much the costs are so they feel more in control there."

The flexibility of private healthcare, which allows patients to move in and out of care pathways, was also a significant factor contributing to the perceived control patients have over their care. This is further influenced by service factors and physician factors.

"You can jump in and out of the private system, so people will use it to get an initial consultation to get a diagnosis quicker, say you have got hip pain, you think it is due to osteoarthritis, you might need a hip replacement, on the NHS locally, you have to go down an MSK pathway, see a physio, maybe try and lose some weight, which could take weeks to months."

The financial status of a patient was a common sub-theme highlighted by both GPs and surgeons that could influence a patient’s decision. A patient’s financial status encompasses aspects such as their occupation, current socioeconomic status, type of insurance plan and the patient’s understanding of the costs associated with private healthcare.

"There is a cost factor to be considered. Whilst I will see a certain percentage of patients coming through company schemes, as they’ve been lucky to be working in a company that offers them insurance… they will understandably come from a higher socioeconomic group."

Some interviewees also noted that the decision to choose between private or NHS healthcare can depend on patient perceptions of both of these services. These can vary and are moulded by aspects such as their expectations of the services based on expenditure, and opinions from other sources. For example, one interviewee highlighted that many patients feel more inclined to choose private healthcare due to a perception of underfunding and cost-cutting within the NHS.

"Everything has always had restrictions. There is a set pot of money provided for all services. There has always been rationing. Money is tight in the NHS and more is rationed so more is not available."

Finally, patient preferences play a large role in helping them decide between the two healthcare systems. These preferences were reported to be primarily developed through prior experiences and satisfaction with the service they are choosing, but also through other factors such as patient perception, physician factors and service factors.

Theme 2: Physician Factors

A major sub-theme that was extensively discussed in many of the interviews was the level of training of the physician.

"You are guaranteeing that the person you see in private healthcare is the person who will be operating on you, you will be operated on by the consultant that you see. Whereas, clearly if you say you have gallbladder problems, and you are referred to a big city, surgical unit, then it may well be the registrar that takes your gallbladder out."

This sentiment of preferring to see consultants as opposed to trainees appeared to stem from different reasons. Some patients preferred to see specific consultants who are prominent in their fields, whereas other patients were reported to not want to be ‘a part of someone’s learning curve'. Interviewees also reported that some patients expected consistency in their care, wanting to see the same physician in all their appointments and seeing the physician who operated on them.

"They do develop a kind of relationship with that person, and I think it helps them if they see somebody who knows them, whereas if they go and see somebody new, then they have to tell the story again, I think it's harder for both parties really."

Another physician-related factor discussed in the interviews was the work environment, which influences a physician's practice and relationship with patients. One interviewee highlighted that private practice often benefits from more effective communication systems between managers and physicians, fostering a more supportive work environment. Additionally, some interviewees noted that smaller healthcare teams in the private sector facilitate closer professional relationships among colleagues. This closer collaboration often leads to improved patient care and higher levels of patient satisfaction, which can subsequently influence patients' future choices between healthcare sectors.

"I think they [private hospitals] are smaller institutions, where you work in a much closer-knit team. I can’t remember operating with the same team this year in the NHS… so you build up a much closer relationship and understanding, and that has been shown to deliver better patient outcomes."

Interestingly, all surgeons reported that their private practice almost mimics their NHS practice in terms of treatment pathways, recommendations and threshold for intervention. On the other hand, GPs reported that there may be more physician autonomy in private healthcare, with fewer restrictions on practice. The surgeons also noted that private healthcare allowed them to take more control of their working hours and schedule.

Theme 3: Service Factors

One sub-theme identified was service delivery, which all interviewees highlighted was a predominant influencing factor. In particular, patients can be seen quicker privately and in a timely manner which may lead them to choose private healthcare over the NHS.

"If people want to go private, it tends to be that they’ll say ‘oh well I’ve got private insurance if that will make it quicker’. I don’t think that they think they get better healthcare going private, to the NHS. I think it purely is a time thing."

Moreover, patients get substantially more time with the doctor during private consultations, allowing a greater time for communication and reducing the risk of confusion leading to an improved doctor-patient relationship and better outcomes.

"To be honest, the more time I spend with patients, they appreciate their condition better. So, I will be able to explain the condition, explain the medication and explain the importance of adherence and compliance to medication as well. Whereas in the NHS, sometimes I don’t have that much time. So, unfortunately, there is time pressure."

Not only is there difficulty in communication with patients due to time constraints, but this difficulty also extends outside the clinic. In the NHS, patients may struggle to get in touch with secondary care clinicians if they have any other queries or questions regarding their care. Privately, however, patients are better able to contact their secondary care clinicians. This problem in communication exists between primary and secondary care as well, making patients feel like the provision of service is disjointed and that they are not receiving ‘continuity of care’. This also ties in with the theme of patient factors as it gives the impression that patients can have more control over their care privately, therefore influencing their perception of the two healthcare systems.

"*I think patients being able to have access to a GP at the start of their journey and to be able to have continuity of care with that GP, I think that’s what patients are missing out and they are being passed around a bit, and they feel like their condition isn’t being addressed properly and they seek private healthcare."*

The service delivery privately also has better infrastructure and hospitality so is able to provide a better healthcare environment and more bespoke care to its patients.

"I think in private hospitals patients like it because it feels very plush, and it feels like they are going into a hotel, it just feels more comfortable. They will have their own room with a TV and an ensuite and that sort of thing, whereas often in the NHS they will struggle because they are on a ward with somebody screaming all night and lights are on and off."

Another sub-theme arising from the analysis is access to service. Guidelines in place for referral to different services, not only restrict physician practices but also limit the patient’s ability to access healthcare services. One interviewee even highlighted barriers that exist to accessing primary care in the NHS given the backlog of patients and understaffing in some practices.

"Patients who want to have an orthopaedic surgery, a routine knee or hip replacement operation will have to be non-smokers and you know BMI below 30 … we have more triaging processes, we have more guidelines to stick to and we also base it on population level needs."

Moreover, the flexibility offered in private healthcare allows patients to move in and out of pathways, where they can get the necessary investigations and diagnosis promptly through private healthcare and return to NHS care and waiting list for treatment.

The final sub-theme within service factors identified in relation to this section is the type of service that the patient is seeking. Almost all interviewees highlighted that certain healthcare services, such as emergency services and complex surgical cases, in the NHS, are better than private healthcare. Some examples discussed were cancer screening and treatment.

"I think of the NHS as kind of providing core medical services in the UK, and excellent multidisciplinary team care."

However, some services are not routinely provided within the NHS and the patient may need to access private healthcare to get this service. Some specialties also have significantly longer wait times and may be particularly difficult to access due to inadequate servicing. Ironically, a number of interviewees suggested that while some services such as cosmetic surgery are not offered within the NHS, a considerable number of patients go privately due to a lack of awareness of what is provided under the NHS as well.

"In the NHS, like you said, things are evolving all the time so it can be very difficult to keep up with what services are available, what is being offered where, who is going to qualify for it. Often, we will refer, and it will get sent back to us saying that it is not appropriate or here is more appropriate."

This lack of knowledge regarding service availability is not only a problem for patients but also for GPs.The GP interviewees noted that the rapidly evolving nature of the NHS means that as more specialties get added, different parts of the system also become privatised making it difficult to be up-to-date with referral pathways. At times GPs are unsure of who the patient has seen and what type of service they have received.

"You have got private companies taking things over, I might be generalising it, but my experience has been where you struggle to find out who the patient has seen, they don’t seem to know who they have seen and you can't find letters or letters haven’t been sent. I have definitely experienced that sort of thing."

The differences in service delivery, type and access between private healthcare and the NHS can be attributed to many factors including understaffing and underfunding within the NHS, as pointed out by many of the interviewees. This creates a perception that the NHS is very limited by these factors which may influence patients to preferentially choose private healthcare.

## Discussion

This study has identified three global themes, namely, patient factors, physician factors and service factors, that influence a patient’s choice between private healthcare and the NHS. A key component of patient factors is demographics. The literature has identified a number of facets within demographics such as age, educational level and ethnicity, among many others, as being important factors in choosing between private or government healthcare services [17,18,19]. For example, older patients or those from a poorer socioeconomic or educational background may take a more passive approach in deciding their healthcare provider, relying more on their GPs to make referral decisions.

With regard to patients’ nature of sickness, complex or chronic healthcare needs have a notable difference in influencing patients’ choices. Patients who suffer from chronic conditions expect a more nuanced relationship with their healthcare provider and more control over their care [17,20]. Our study identified patient perceptions and preferences as a patient factor in decision-making. This finding is supported by the literature, which discusses aspects such as lack of information about their healthcare provider, the reputation of a hospital, their relationship and communication with certain doctors, previous negative experiences and the anticipation that junior doctors or students may be part of their care, being important factors in their decision making [17,18,19,20,21].

When considering physician factors, this study identified two distinct sub-themes: the level of physician training and the environment the physician works in. The participants in this study noted that some patients are hesitant to be seen by junior doctors, with one participant saying ‘patients don’t want to be a part of someone else’s learning curve’. The literature shows that a large majority of patients think that it is important to know the level of training of their doctor [21]. This extends to medical students, whereby patients are more comfortable with senior-year medical students being present at intimate examinations [22]. Ironically, most patients do not know the level of training of their doctors [23]. While patients are largely supportive of medical training and view junior doctors and medical students as an integral and positive aspect of healthcare, a considerable number of people still prefer to go to non-teaching hospitals [24]. This could be because of the wide range of terms used for doctors at different levels of their training (such as junior doctor, house officer, registrar, specialty doctor and trainee). This poses a risk of patients being confused about the training and hierarchy of doctors within the NHS and hence may contribute to misperceptions of junior doctors being underqualified to deliver a certain standard of healthcare. Junior healthcare providers, although competent in clinical skills and knowledge, may lack certain soft skills such as empathy and communication, which enhance patient care [21,22,23,24]. When considering the work environment of physicians, this study was able to identify how environments affect the way in which physicians work and why this indirectly impacts the delivery of patient care. Studies have shown that working in private healthcare can provide a more relaxed and controlled work environment with flexibility, empowering them to deliver a more efficient healthcare service, thus improving patient experience [25,26]. Despite the benefits of working privately, many doctors remain sentimentally attached to the NHS and acknowledge the potential detrimental impacts of private healthcare on the NHS [26]. This is supported by Goodair et al., who demonstrated an increase in treatable deaths in the UK associated with increased outsourcing of NHS services to private healthcare [27]. Nevertheless, there is a lack of quantitative evidence looking into the impacts of private healthcare on the NHS and therefore more research within this field is crucial.

When discussing service factors, three sub-themes have been noted: service delivery, type of service and access to service. Service delivery is a broad sub-theme covering aspects such as waiting times, continuity of care, communication provided by the service and the environment and infrastructure of the service. While waiting times are a significant problem in the NHS, with ample amounts of evidence to support its negative impacts on patient care [20,28,29], other facets of service delivery such as the amenities and facilities, cleanliness and the general reputation have all been identified as equally important [18]. The type of service that the patient is seeking is identified as another sub-theme of this study that contributes to patient decisions between public and private healthcare. This is supported by other studies that conclude that service unavailability pushed patients toward seeking alternate options [18]. Moving on to discussing access to services, participants of this study noted that secondary care in the NHS is more difficult to access due to complex or strict referral pathways. Integrated care pathways that encompass primary and specialist care providers hold the potential to streamline and enhance patient care. However, the successful implementation of such pathways requires commitment across multiple organisations, robust IT capabilities and financial support [30].

While the three global themes were distinctly identified in this study, there are nuanced overlaps within these themes, which were observed in the study and corroborated by the literature in the field.

Limitations of the study

There are two main limitations to this study. Firstly, the study has only 10 participants from one affiliated institution (HYMS). While the last two interviews did not identify any new themes, the sample size and geographical limitations of the sample mean that readers should be aware of the limitations of transferring the findings to other healthcare systems. Secondly, the study uses physicians to identify patient factors and hence may not have accurately captured patient factors. However, this study did not adequately analyse the barriers posed by factors outside of the service, such as the geographical location of the service among others [18].

## Conclusions

This study was able to highlight physician perspectives on three global themes: patient, physician and service factors. Although these themes had significant overlap, their nuanced interactions warrant more in-depth analysis. The significance of these factors may vary between patients, but this study provides a more physician-centred perspective on the complex relationship between the NHS and private healthcare and fills a key gap in the literature. While the study had several limitations, it provides a foundation for further research into factors that influence a patient's decision in selecting their healthcare provider. 

## Figures and Tables

**Figure 2:**
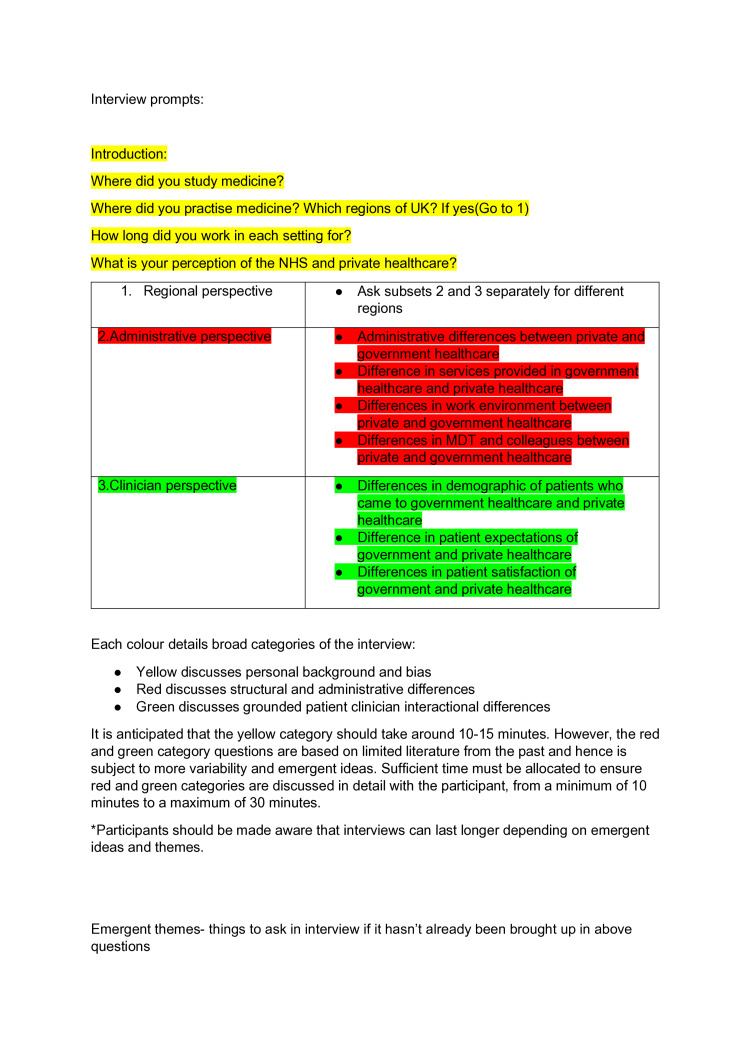
Study interview prompt (part 1)

**Figure 3:**
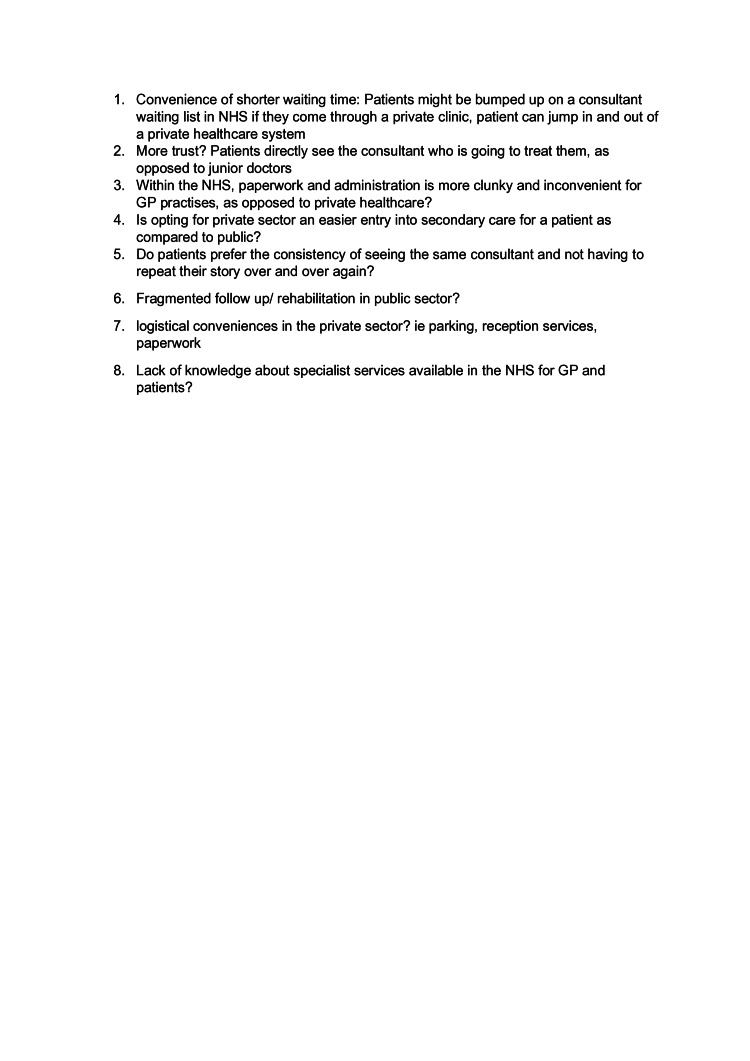
Study interview prompt (part 2)

## Appendices

Study interview prompt
